# Ambition or comparison? Socioeconomic status and wellbeing differences between local and migrant workers

**DOI:** 10.1371/journal.pone.0289092

**Published:** 2023-07-27

**Authors:** Dan Li, Xiaocong Yang, Guanyang Zou

**Affiliations:** 1 School of Management, Institute for Population and Social Policy Studies, Xi’an Polytechnic University, Xi’an, China; 2 School of Public Administration, Guangzhou University, Guangzhou, Guangdong, China; 3 Nossal Institute for Global Health, School of Population and Global Health, Melbourne, Victoria, Australia; 4 School of Public Health and Management, Guangzhou University of Chinese Medicine, Guangzhou, Guangdong, China; Centre for Demographic Studies, SPAIN

## Abstract

Pursuing wellbeing is an essential part of human life and plays a determining role in public health and social sustainability. Prior research identified objective socioeconomic status (O-SES), such as real income and homeownership, as facilitators of human subjective wellbeing (SWB). However, not all humans with better SES reported high SWB. This paper expects that subjective socioeconomic status (S-SES) is the key path through which O-SES shapes SWB and that this indirect relationship varies by household registration status, length of residentship, and type of migrant status. Based on a national representative survey dataset-China General Social Survey 2010 (CGSS), the results of generalized structural equation modeling (GSEM) show that household income and homeownership as O-SES are positively related to SWB. Self-evaluated household SES as an important indicator of S-SES not only has a positive relationship with SWB but also significantly mediates the relationship between O-SES and SWB, especially for the new-local residents (NLRs), urban-to-urban migrants (UUMs) and rural-to-urban migrants (RUMs). This study has substantial implications for targeting the comparative psychology and sustainable productivity of Chinese migrants and the local labor force since it is currently facing a growing aging society.

## 1. Introduction

Psychological wellbeing, as the broadest term which includes subjective wellbeing (SWB), is an essential psychological indicator that social scientists and psychologist frequently utilize for public health and social stability [1]. Conceptualized as multifaceted with affective and cognitive components, SWB reflects individuals’ satisfaction with life quality [2, 3]. The accumulated evidence show that not only is SWB influenced by an individual’s health, but it also contributes to health outcomes in reverse [1, 4]. The lower level of SWB and the enormous gap in SWB between advantaged and disadvantaged groups may stir up a higher crime rate, social unrest, and lower economic growth [3, 5].

Objective socioeconomic status (O-SES) indicated by income level and homeownership, is usually used to predict respondents’ happiness. Some studies discovered that reported happiness are likely to benefit from improved O-SES [6]. This may be attributed to that people often judging whether a person is successful by their wearing, living place, vehicle brand and other people’s evaluation in current social context. People who are considered success tend to accept more respect and approval, which increases their happiness to some degree.

However, few studies have emphasized the importance of subjective socioeconomic status (S-SES) for SWB. S-SES, which is indicated by self-evaluated socioeconomic status (SES), aspiration income, the difference between aspiration income and absolute income, is found to be positively related to SWB. Subramanian [7] holds the point that S-SES did have an indirect and positive relationship with SWB through life satisfaction because SWB requires one to be content with the reality of his/her life. To some degree, S-SES (versus O-SES) plays a more important role in SWB, and the mediating effect of S-SES may weaken the positive relationship between O-SES and SWB.

The relationship between O-SES, S-SES, and SWB is worth investigating in-depth, especially for Chinese migrants. With the reform and opening-up policy and joining the World Trade Organization (WTO), China is confronted with a labor supply shortage that stimulates increasing migrant workers. The rise of the Chinese construction, manufacturing, textile, and financial industries has created a “migration wave”. Not only is the scale of rural-to-urban migrants (RUMs) overgrowing, but more urban (nonagricultural) Hukou individuals would also migrate to economical metropolitan cities that could be named “urban-to-urban migrants (UUMs)”. Due to the resource isolation and barriers caused by the household registration system in the early stage, people in different regions enjoy different resource conditions, such as educational resources from childhood. Therefore, respondents with different Hukou background differs from each other in terms of pressure, requirements of income, living standards, pursuit, career aspirations and perception of SWB in the urban labor market, especially in the first-tier cities. It is necessary to take the difference of each group into account and classified all the respondents in depth to explore the SWB for each group. Regarding Chinese national conditions, UUMs are generally more educated than RUMs, and are likely to be engaged in higher status occupation categories than RUMs as well [8]. In addition, there is another kind of “migrants” living in the host location who are classified as local residents. These people moved their household registration place to the host city based on the length of residence, educational background and occupation, who are called new-local residents (NLRs) in the host city playing an indispensable role in city construction, sustainable economic growth and country development as well. There are approximately 493 million people (35% of the total Chinese population) whose workplace is not the same as their household registration location [9]. However, the vast scale of labor force migration exposed inequitable treatments in the Chinese labor market, such as inequality of income, job opportunities, social security and other forms of welfare [3, 10]. Although most migrants are aware of the unequal treatment in nonlocal urban cities, the number of migrants to the more developed region is still growing, especially UUMs because there are more job opportunities and higher income in the metropolis. However, such unequal treatment could give rise to depression of these migrants concerning their daily life and the process of seeking job [11]. Therefore, migrants may report lower levels of SWB than local residents. As migrants serve as the basic building blocks of city construction and economic development in China, their SWB not only play a crucial role in their physical and psychological health status but are also closely associated with Chinese stability and development. To conclude, this research focuses on Chinese migrants’ SWB and explore how O-SES and S-SES relate to their SWB.

This study contributes to the wellbeing literature from the following aspects. First, not only O-SES and S-SES will affect SWB at the same time, but different measurements of S-SES (e.g., personal ideal aspirations and comparison psychology) also exist simultaneously in reality. Therefore, we analyze the commonalities of related theories about the definitions and measurements of S-SES, and integrate them into multiple measurements of S-SES, for example, the Social Comparison Theory (SCT) and the Aspiration Level Theory (ALT). This research extends the previous studies by taking into account the existence of multiple measurements of S-SES simultaneously when investigating the mediating effect of S-SES on the relationship between O-SES and SWB. Second, as people with different Hukou backgrounds differ in terms of requirements, lifestyle and personal goals, this study goes beyond earlier research by distinguishing all the respondents in-depth into NLRs, native residents (NRs), UUMs and RUMs. Overall, this study focuses on the following three research questions: 1) the impact of O-SES and S-SES on SWB; 2) whether O-SES affects SWB through S-SES; 3) whether the influence of O-SES and S-SES on SWB varies based on respondents’ Hukou status, length of residency, and type of migrant status. In order to deal with these research question, this study proposes corresponding hypotheses. The following section presents our review of the literature and hypothesis. Section 3 describes the data, variable selection and methodology in our analysis. Section 4 interprets the empirical results, followed by the final section of results discussion, conclusion, limitation of this study, viable further research, and policy implications.

## 2. Literature review and hypothesis development

### 2.1 Objective SES (O-SES) and subjective wellbeing (SWB)

**Fig 1:**
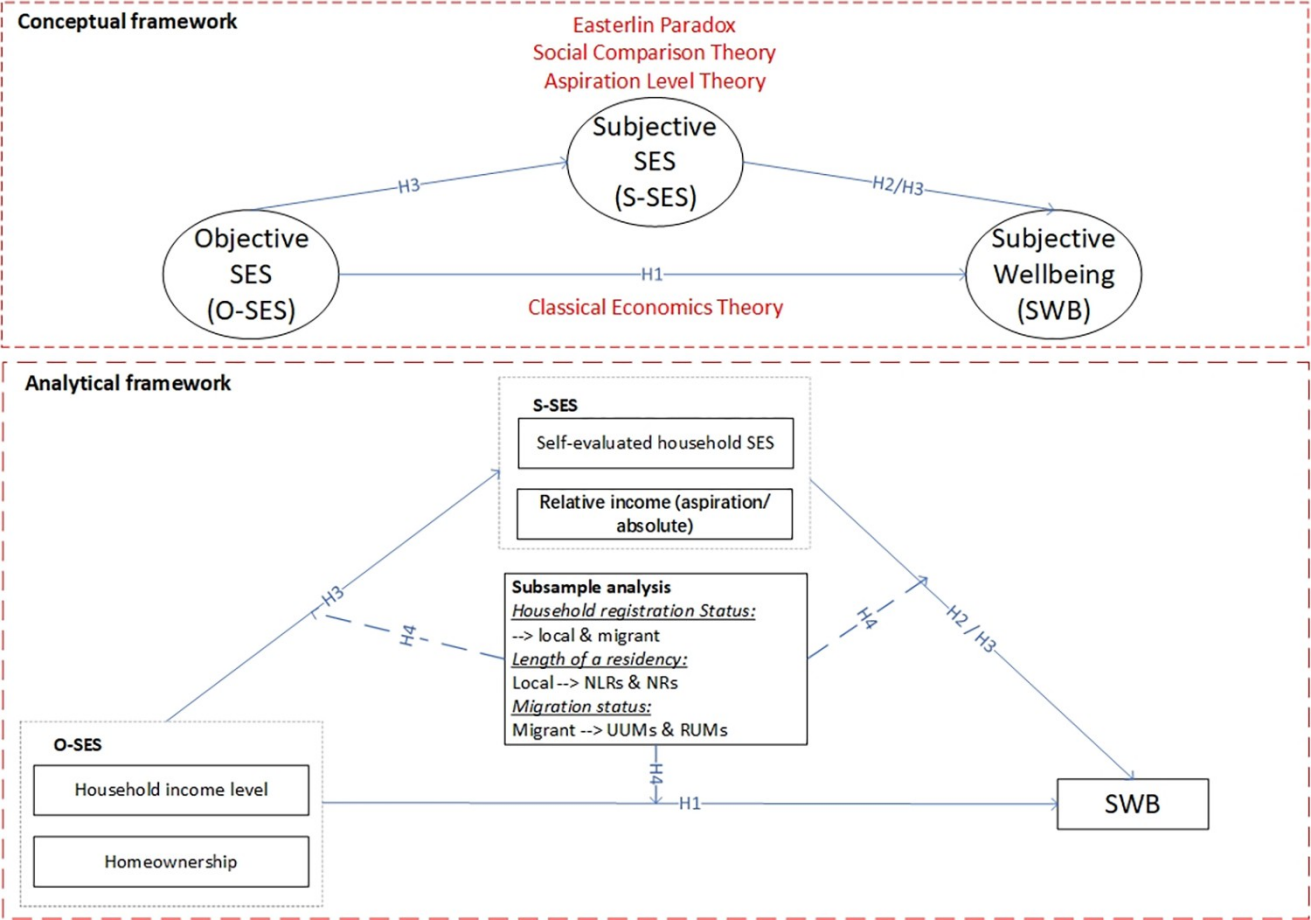
Theoretical and analytical framework. Notes: 1) GSEM: generalized structural equation modeling, O-SES: objective socioeconomic status, S-SES: subjective socioeconomic status, SWB: subjective wellbeing; 2) Solid lines are full sample analysis while dashed lines are subsample analysis.

The analysis of SWB has been a rapidly growing topic for sociologists, psychologists, and economists over recent decades. However, there remains no consensus on the determinants of SWB. Regarding the incipient cognition of SWB, most studies proposed the linkages between individuals’ O-SES and SWB from the aspects of income and the homeowner status [12, 13]. O-SES refers to an individual’s social and economic position in society based on quantifiable and external factors which is usually utilized as an indicator of access to power and resources, and also have a significant influence on individual’s SWB [14–16]. Homeowner status refers to respondents who own property in the city where they work or live [16]. The reason why homeowner status is usually considered as an O-SES is attributed to its strong connection with wealth accumulation and overall financial stability for families [17, 18]. Homeowner status encompass a greater amount of information, as they do not only reflect an individual’s economic standing but also their social status. Moreover, for local residents who are the crucial control group in this study, their longer duration of residence in the host city allows them to accumulate greater social networks and financial resources [19, 20]. They can inherit properties through intergenerational transfer [20, 21]. Therefore, even though many local residents may have less educational attainment, they still possess a sense of superiority over migrants and enjoy higher social status. Additionally, the profound influence of China’s millennia-old traditional culture can lead the majority of individuals to attach great importance to the notion of “home” in their mindset [22]. Within most households, real estate constitutes a significant proportion of their financial structure. As a result, the ownership of property in the host city serves as an indication of one’s sense of belonging and indirectly validates their social status within the community [21, 22]. Taking into account the various reasons mentioned above, homeowner status appears to be a more suitable representative variable for O-SES in the Chinese context. They perceived that economic growth indicated positive changes in human happiness [23]. Material requirements could be better satisfied through higher income and, therefore, positively associated with individuals’ SWB. Few studies on SWB showed that homeownership positively contributed to individuals’ happiness, as owning a property could reduce the anxiety caused by the lack of a settled home and the uncertainty of housing rent. In contrast, individuals living in rental houses are likely to have a poor feeling of residential satisfaction and security [22, 24]. As shown in Fig 1, according to Classical Economic Theory (CET), it is likely that O-SES is positively related to SWB [25]. Therefore, we propose the following hypothesis:

**Hypothesis 1**: Respondents’ income and homeownership status as O-SES is positively related to SWB, respectively.

### 2.2 Subjective SES (S-SES) and subjective wellbeing (SWB)

In contrast, the well-known “Easterlin Paradox” was been proposed [26], pointing out that SWB is not correlated with respondents’ O-SES but is associated with their S-SES [26–28]. S-SES generally refers to the individual’s economic status psychologically identified by comparison with reference objects.

Based on the “Easterlin paradox”, a growing number of studies explore the association between SWB and S-SES [27, 29]. As shown in Fig 1, there are many methods to measure S-SES. Following SCT, the most popular measurement of S-SES is the relative income that respondents evaluate themselves to assess their own lore status on a wide range of variables [30]. A significant issue arising from S-SES is which reference group they generally compare with. The common reference group is likely to be respondents’ neighbors, colleagues, and peers. Some researchers suggest that if respondents reside in an affluent area where neighbors earn a higher wage, their consumption pattern will be upward influenced [27, 29, 31]. Stimulated by comparing psychology, respondents’ consumption standards may be enhanced upward correspondingly. The long-term excessive financial burden will eventually reduce the SWB of respondents. In addition, respondents will self-evaluate their SES by comparing the O-SES with other familiar individuals (e.g., colleagues, peers) or the average level of community, city or country into different social hierarchies, e.g., much below average, below average, average, above average and much above average [5, 31]. It is found that SWB rely positively on their S-SES [5]. However, this positive relationship only holds for the wealthy person rather than the poor [32]. For poor individuals whose SES is below average, SWB was negatively associated with the difference between their absolute income and average level. The larger the gap between the actual income and the average income of the whole society, the lower the levels of SWB will be.

Another popular measurement of S-SES is through psychological aspiration income, which relies on ALT. According to ALT, the association between SWB and aspiration suggests that one’s psychological desire, consumption behavior, and requirements of living standards increase correspondingly with earnings growth. Aspirations of higher material standards may counterbalance the positive impacts of absolute income increases on individuals’ SWB in the long-term [33]. The SWB mostly depends on the difference between aspirations and actual achievement [34, 35]. Following the ALT, the pure impact of aspiration income and absolute income on SWB was been examined by Stutzer [34]. It is found that the difference between aspiration and absolute income (the ratio of aspiration income to absolute income) matters more for SWB significantly. Moreover, the more considerable discrepancy between aspiration income and absolute income contributes to a lower level of SWB. Therefore, based on the commonalities of the above theories about S-SES in terms of definition and measurements, we integrated these theories to construct our analysis model and proposed the following hypothesis:

**Hypothesis 2:** Following SCT, individual’s self-evaluated SES as S-SES is positively related to SWB, while, based ALT, the difference between aspiration income and real income as S-SES is negatively correlated to respondents’ SWB.

### 2.3 The mediating role of S-SES in the relationship between O-SES and SWB

At present, most previous research has weakness that it treated O-SES and S-SES as two opposite theories and utilized a “black or white” attitude to explore which theory contributes to people’s SWB [27, 28]. However, O-SES and S-SES almost impact SWB simultaneously in reality, as S-SES is a self-psychological evaluation based on one’s own O-SES. In addition, different measurements of the S-SES can influence the psychological perception of SWB simultaneously as well. Thus, O-SES usually has an indirect contribution to SWB by acting on the different measurements of S-SES. Such a complicated relationship calls for an investigation of the mediating effect of S-SES on the relationship between O-SES and SWB. This article will fill the gap of previous studies by combining the related theories together to construct a mediating effect model which includes O-SES and S-SES simultaneously to examine their relationships with SWB. Consequently, Hypothesis 3 is proposed:

**Hypothesis 3:** S-SES mediates the positive relationship between O-SES and SWB, and the mediating effect of S-SES will weaken the original relation between O-SES and SWB.

### 2.4 The heterogeneous effect analysis

Another weakness of previous studies revolves around sample selection. Most studies mainly divided the total sample into local Hukou residents and nonlocal migrants. Migrants are only concerned about RUMs and estimate the determinants of their happiness [36]. UUMs, as an indispensable part of migrants, are usually neglected in the sample. The motivation for migrating between RUMs and UUMs are not precisely the same in China. As discussed before, the Hukou system results in the resource isolation and barriers between different regions in the early stage. Respondents with different Hukou background certainly have different requirements of income, personal goals, lifestyle, aspirations and perception of PWB in the urban cities, especially in the metropolitans. Consequently, it is unreasonable to ignore the difference between UUMs and RUMs, and we should classify the labor force in Chinese urban labor market in more detail on the basis of Hukou status. The purpose of RUMs is to earn money and send it back to improve the living conditions for their family members rather than to be rooted in metropolitan cities [37]. In contrast, UUMs usually have more educational attainment than their rural counterparts. Most of them choose to stay in a large city where they graduated from university. Moreover, many UUMs are driven by great ambition to metropolis even if they understand the unfair treatments in unfamiliar cities [38]. They expect to settle down through going to university and working. Therefore, there are huge differences between UUMs and RUMs in terms of educational background, purpose and expectations, and UUMs, as an essential portion of the migrant group, should not be ignored. In addition, some migrants who are most likely to be UUMs have obtained local Hukou identity and become NLRs due to their academic qualifications, occupation type, and social security payment. Such NLRs are essentially transformed from migrants to local residents. However, most previous articles simply classified NLRs into native groups based on Hukou status, which will result in biased estimation. Therefore, in addition to dividing the total sample into local residents and migrants, it is also necessary to classify these two groups in detail. According to the length of residential difference, all local residents can be divided further into NRs who have lived in the host city since birth and NLRs who migrated to the host city and obtained the local Hukou identity later. Based on the type of Hukou status, migrants can also be divided into UUMs and RUMs. Thus, we propose the following hypothesis:

**Hypothesis 4:** The mediating effect of S-SES on the relationship between O-SES and SWB varies by respondents’ Hukou status (local residents versus migrant sample), length of residency (NRs versus NLRs), and type of migrant status (UUMs versus RUMs).

## 3. Methods

### 3.1 Sample

We utilized data from the China General Social Survey (CGSS) for our study. The CGSS is a comprehensive database that captures data from various societal levels—individuals, families, and communities—and outlines social change trends. The 2010 CGSS dataset, in particular, offers a nationally representative sample in China, enabling us to generate persuasive findings. This period, marked by rapid growth and urbanization in China, was devoid of policy restrictions, such as housing purchase limitations, that could potentially disrupt population mobility and bias our research [39, 40]. Conversely, post-2010 saw the introduction of such policies, coupled with a significant surge in housing prices in 2012, which could impede migrant wellbeing [40–45]. Therefore, our choice of the 2010 CGSS dataset ensures the robustness of our study and minimal policy interference. Moreover, it provides access to individual aspiration income—a crucial component of S-SES—which is exclusively available in this year’s survey, and allows us to avoid the impact of post-2010 housing purchase restrictions and price surges.

The total sample can be divided into NRs, NLRs, UUMs and RUMs based on their Hukou status and length of residential difference. Specifically, this study’s sample (see Table 1) is restricted to 442 migrants (233 UUMs and 209 RUMs) and 4,504 local Hukou individuals (3,722 NRs and 782 NLRs). While the overall sample size (4,960) and the sub-sample size (442) may appear to be relatively small at first glance, it is important to note that as a nationally representative sample, the stratified and reliable sampling method used has been validated extensively in numerous studies for its reliability and representativeness [46–51]. Furthermore, in the field of social science research, empirical analysis results from an overall sample exceeding 1,000 observations and sub-samples exceeding 100 observations typically do not differ statistically significantly from the analysis of the population sample [52–54]. Lastly, we conducted a post-hoc power analysis to ascertain the statistical power of our study given the sample size. The results indicate that our study boasts a power of 0.8012, generally accepted as satisfactory in social science research [55, 56].

**Table 1:** Variable statistics description, full, migrants and local subsample sample.

Variables	Migrants	Local resident	Total	
	(N = 442)	(N = 4,518)	(N = 4,960)	p-value
**Dependent variable**				
Subjective wellbeing				0.466
Very unhappy	9 (2.0%)	80 (1.8%)	89 (1.8%)	
Unhappy	27 (6.1%)	308 (6.8%)	335 (6.8%)	
Neutral	89 (20.1%)	797 (17.6%)	886 (17.9%)	
Happy	261 (59.0%)	2647 (58.6%)	2908 (58.6%)	
Very happy	56 (12.7%)	686 (15.2%)	742 (15.0%)	
**O-SES**				
Household income per capita (HIPC)				<0.001
Mean (SD)	50564.67 (149743.70)	15871.62 (39929.03)	18963.22 (59531.65)	
Min, Max	0.0, 2800000.0	0.0, 2000000.0	0.0, 2800000.0	
Homeowner of current apartment/house				<0.001
No	267 (60.4%)	1178 (26.1%)	1445 (29.1%)	
Yes	175 (39.6%)	3340 (73.9%)	3515 (70.9%)	
**S-SES**				
Relative income (aspiration/absolute)				0.078
Mean (SD)	2.38 (3.44)	3.03 (7.64)	2.97 (7.37)	
Min, Max	0.0, 40.0	0.0, 250.0	0.0, 250.0	
Self-evaluated Household SES				0.241
Low-level	156 (35.3%)	1733 (38.4%)	1889 (38.1%)	
Medium-level	235 (53.2%)	2357 (52.2%)	2592 (52.3%)	
High-level	51 (11.5%)	428 (9.5%)	479 (9.7%)	
**Control variables**				
Location				<0.001
Rural	0 (0.0%)	2085 (46.1%)	2085 (42.0%)	
Urban	442 (100.0%)	2433 (53.9%)	2875 (58.0%)	
Hukou status				0.074
Non-agriculture	209 (47.3%)	1937 (42.9%)	2146 (43.3%)	
Agriculture	233 (52.7%)	2581 (57.1%)	2814 (56.7%)	
Length of resident				<0.001
Mean (SD)	6.98 (7.74)	37.59 (13.88)	34.86 (16.03)	
Min, Max	0.0, 52.0	0.0, 60.0	0.0, 60.0	
Length of Hukou registered				0.001
Mean (SD)	34.96 (9.68)	37.22 (14.23)	37.02 (13.90)	
Min, Max	18.0, 60.0	0.0, 60.0	0.0, 60.0	
Employment status				<0.001
Unemployed	7 (1.6%)	154 (3.4%)	161 (3.2%)	
Farm	5 (1.1%)	1506 (33.3%)	1511 (30.5%)	
Self-employed	119 (26.9%)	732 (16.2%)	851 (17.2%)	
Employed	311 (70.4%)	2126 (47.1%)	2437 (49.1%)	
Ownership of employer				0.005
Private	402 (91.0%)	3896 (86.2%)	4298 (86.7%)	
Public	40 (9.0%)	622 (13.8%)	662 (13.3%)	
Annual working hours				0.258
Mean (SD)	2696.94 (1016.30)	2636.89 (1070.98)	2642.24 (1066.26)	
Min, Max	52.0, 6552.0	52.0, 8736.0	52.0, 8736.0	
Age				<0.001
Mean (SD)	34.96 (9.68)	41.82 (9.85)	41.21 (10.02)	
Min, Max	18.0, 60.0	18.0, 60.0	18.0, 60.0	
Gender (M = 1; F = 0)				0.864
Female	189 (42.8%)	1951 (43.2%)	2140 (43.1%)	
Male	253 (57.2%)	2567 (56.8%)	2820 (56.9%)	
Marital status				<0.001
Single/separate/divorce	114 (25.8%)	543 (12.0%)	657 (13.2%)	
Married/cohabit	328 (74.2%)	3975 (88.0%)	4303 (86.8%)	
Educational level				<0.001
Illiteracy	13 (2.9%)	392 (8.7%)	405 (8.2%)	
Compulsory	168 (38.0%)	2457 (54.4%)	2625 (52.9%)	
Senior high school	107 (24.2%)	904 (20.0%)	1011 (20.4%)	
College/University	154 (34.8%)	765 (16.9%)	919 (18.5%)	
Self-rated Health				<0.001
Poor & worst	20 (4.5%)	556 (12.3%)	576 (11.6%)	
Neutral	79 (17.9%)	942 (20.8%)	1021 (20.6%)	
Good	162 (36.7%)	1618 (35.8%)	1780 (35.9%)	
Excellent	181 (41.0%)	1402 (31.0%)	1583 (31.9%)	
Social security				<0.001
Otherwise	117 (26.5%)	361 (8.0%)	478 (9.6%)	
Covered by social security	325 (73.5%)	4157 (92.0%)	4482 (90.4%)	
Number of children				<0.001
Mean (SD)	0.96 (0.84)	1.50 (0.94)	1.45 (0.95)	
Min, Max	0.0, 4.0	0.0, 8.0	0.0, 8.0	
**Instrumental variables (IVs)**				
Average HIPC (RMB) at county/city level				<0.001
Mean (SD)	27031.10 (19969.47)	14289.88 (11343.25)	15425.29 (12878.04)	
Min, Max	3411.1, 120994.7	3227.9, 120994.7	3227.9, 120994.7	
% homeowners within the city/county				<0.001
Mean (SD)	60.36 (13.65)	68.62 (11.06)	67.88 (11.55)	
Min, Max	23.9, 92.6	23.9, 92.6	23.9, 92.6	
Gini coefficient at city/county-level				0.108
Mean (SD)	0.47 (0.12)	0.48 (0.09)	0.48 (0.10)	
Min, Max	0.3, 0.8	0.3, 0.8	0.3, 0.8	
% individuals have income in a county/city				0.001
Mean (SD)	91.17 (6.07)	90.11 (6.63)	90.20 (6.59)	
Min, Max	74.3, 100.0	69.1, 100.0	69.1, 100.0	

Source: CGSS 2010.

Notes: 1) Respondents are labor forces aged 16–60; 2) for continuous variables the mean and standard deviation (in parentheses) are presented, while for categorical variables the number of respondents and percentage of the sample (in parentheses) are presented; 3) for continuous variable the analysis of variance (ANOVA) has been used while for categorical variables the chi-square test has been used to show the between-groups-difference, and Sig: * p<0.1, ** p<0.05, *** p<0.01.

### 3.2 Measures

#### Subjective wellbeing

As shown in previous research [16, 57–59], individuals were instructed to indicate the extent to which they feel happy on a five-Likert point scale (ranging from 1 to 5 with “very unhappy” to “very happy”).

#### Objective SES (O-SES)

In Chinese culture, family occupies a very important position and family values is also emphasized. The consciousness of family is prior to the individualism; thus, the economics status in this study is measured in households. O-SES was measured using two indicators, namely, household income per capita (HIPC) and homeownership status, which reflect respondents’ social and economic absolute position [16].

#### Subjective SES (S-SES)

Based on SCT and ALT [5, 32, 60], we use self-evaluated household-level SES (i.e., SCT), the difference between an individual’s aspiration income and absolute income (aspiration income/absolute income) to measure the S-SES (ALT) in our research [34, 35, 61].

Specifically, one item was used to measure household-level SES (i.e., which level is your household SES at?). The original 5-point Likert scale of household-level SES was recoded into three main categories: “lower status” (= 1, 2), “medium status” (= 3) and “higher status” (= 4, 5).

#### Control variables

We controlled for individuals’ age (the Labor Law of the People’s Republic of China stipulates that the working age for male is 18–60 and 18–55 for female, thus, age from 16 to 60 for males and 16 to 55 for females are our main respondents), gender, marital status, length of residence, location, length of Hukou register, self-rated health status (SRH), educational attainment, social security insurance, employment status, annual working hours, ownership of employer, and number of children because these variables were related to wellbeing.

#### Instrumental variables (IVs)

This study acknowledges the possibility of endogeneity, which may infuse bias into model estimation. This issue typically arises when independent and mediating variables are subject to measurement errors or correlate with the error term in the model. To counteract the potential bias arising from endogeneity, we employ instrumental variables (IVs) in our model. Our choice of IVs is guided by previous research and the availability of data for our investigation [62–64]. Specifically, the following four variables have been selected as our IVs: 1) average per capita household income at the county or city level (IV for HIPC), 2) the percentage of homeowners in a county or city (IV for individual homeowner status), 3) the Gini coefficient at the county or city level (IV for self-reported household SES), and 4) the percentage of individuals earning an income in a county or city (IV for relative income).

Before incorporating these IVs into our Generalized Structural Equation Model (GSEM) estimation, we first examine their relevance to our objective socioeconomic status (O-SES) and Subjective Socioeconomic Status (S-SES), and their exogeneity with respect to the error term and SWB in the estimation model. Assuming SWB as a continuous, rather than ordered variable, we conduct a two-stage least squares (2SLS) regression using the instrumental variables separately. Our results confirm that all four IVs meet both the relevance and exogeneity conditions. In the first-stage regression, the F-statistic exceeds 10 and is statistically significant (p < 0.01) for each estimation involving different IVs, demonstrating a strong correlation with our endogenous variables, O-SES and S-SES, and thereby solidifying their position as robust IVs. Furthermore, the p-value for both the Sargan test and the Hansen J test exceeds 0.1, indicating these variables are independent of the error term and are therefore valid instrumental variables. In essence, these four macro-level IVs do not exhibit a statistically significant correlation with SWB.

### 3.3 Ethics statement

We acquired authorization to utilize the publicly accessible CGSS. Access to this dataset is granted upon registration. The ethics committee of the CGSS team, the Renmin University of China, and the Hong Kong University of Science and Technology are responsible for ethics approval and consent to participate. All personally identifiable information was removed upon receiving the data. Consequently, this study no longer necessitates ethics approval or informed consent.

### 3.4 Statistical analysis

We utilize GSEM with ordinal SWB to explore its relationship with O-SES and S-SES as the mediating effect analysis by GSEM can make up for the shortcomings of traditional regression and GSEM enables simultaneous estimation of all parameters in the model. The reason that the traditional regression analysis is not employed, as both the mediating variable S-SES and O-SES in this paper are more than one and the analysis process and the interpretation of our results will be very challenging based on OLS. In addition, the standard error in estimating the mediating effect is large and the coefficient “a×b” obeys a normal distribution when applying the Sobel test under the regression analysis [65, 66]. The advantage of employing GSEM in our study includes the following: (1) it combines the advantage of estimation power and flexibility from both structural equation modeling and a general linear model; (2) it not only allows us to estimate the direct effect of O-SES on SWB but also allows us to estimate the mediation effect of S-SES on the relationship between O-SES and SWB separately and simultaneously; and (3) it can provide less-biased estimation for the right-skewed distribution of the outcome variable of SWB and discontinues mediators of S-SES (i.e. self-evaluated household SES). However, the disadvantage of GSEM is also obvious. Unlike SEM, GSEM cannot perform a goodness of fit test, and any adjustment or modification method is not able to apply to it directly. To overcome these shortcomings, we use the indexes of -2Log pseudolikelihood, Akaike Information Criterion (AIC) and Bayesian Information Criterion (BIC) in stepwise analysis to show the model improvement.

**Fig 2:**
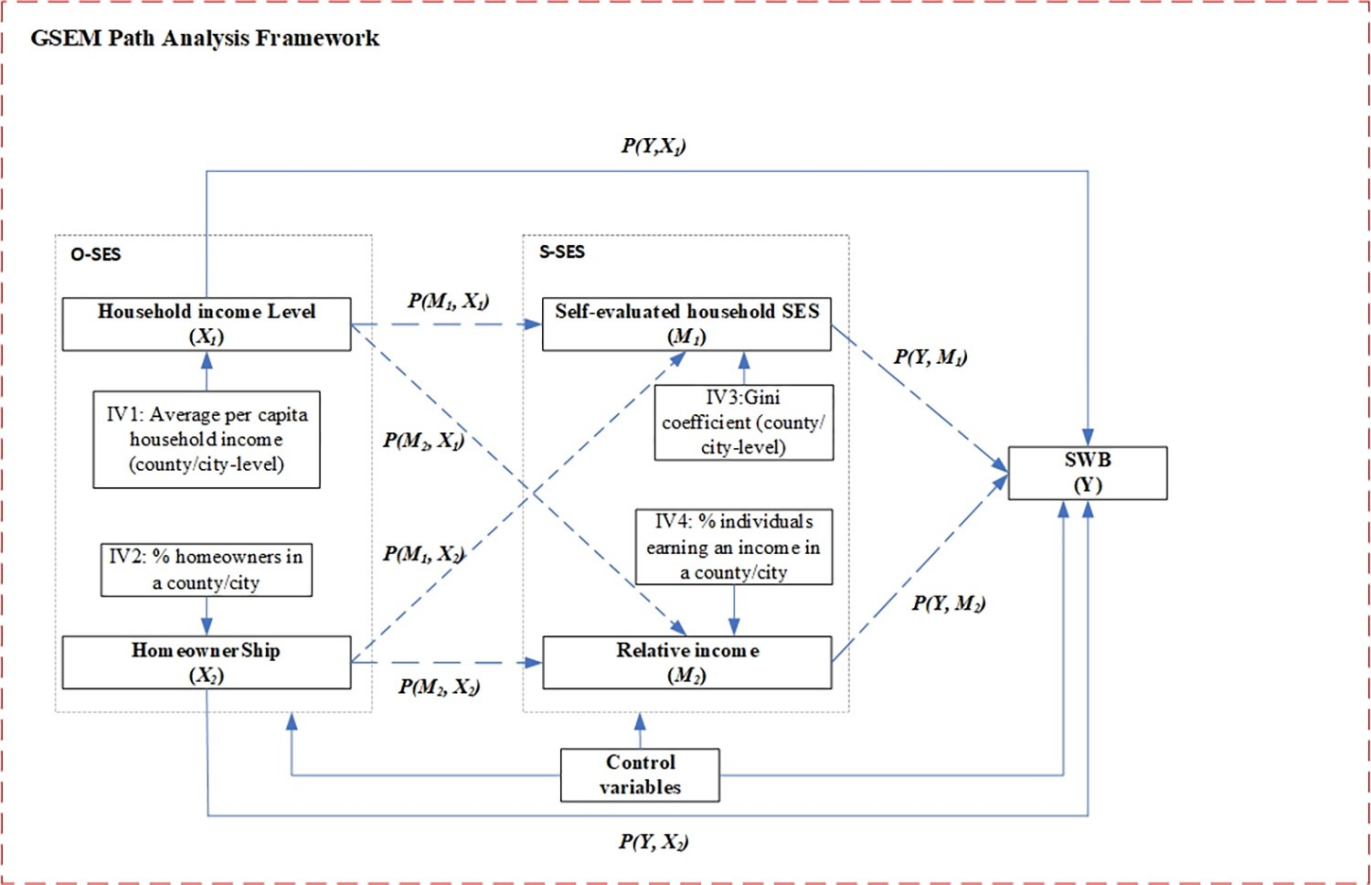
GSEM path analysis framework. Notes: 1) GSEM: generalized structural equation modeling, O-SES: objective socioeconomic status, S-SES: subjective socioeconomic status, SWB: subjective wellbeing; 2) Solid lines are direct effects and dashed lines are indirect effects; 3) each IV was controlled for each O-SES and S-SES variable, while control variables were included across all analysis.

This study utilizes path analysis in its modern GSEM framework (see Fig 2). All the independent variables and mediators are linked by straight arrows that indicate the directions of the relationships between them. In Fig 2 above, household income level (X_1_) and homeownership (X_2_) are assumed to be exogenous and not correlated after controlling the valid IVs mentioned above, while the mediating variable (S-SES) are endogenous. The pathway in the structural equation model that would describe the relationship between household income (X_1_) and SWB (Y) is:

$$r_{\left(Y,X_{1}\right)}=p_{\left(Y,X_{1}\right)}+p_{\left(M_{1},X_{1}\right)}*p_{\left(Y,M_{1}\right)}+p_{\left(M_{2},X_{1}\right)}*p_{\left(Y,M_{2}\right)}$$ (1)

where *r* is the correlation coefficient from a standard correlation matrix containing all the variables in the model and *p* as the path coefficient is the standardized beta coefficient from the regression model. The generalized structural equation indicates that the total correlation between all variables, is the sum of each possible pathway that connect those two variables. In the example of Eq (1), $p_{\left(M_{1},X_{1}\right)}$ refers to the proportion of the variance accounted for by the direct pathway between self-evaluated household SES (*M*_*1*_) and household income (*X*_*1*_), while $p_{\left(M_{1},X_{1}\right)}*p_{\left(Y,M_{1}\right)}$ is the proportion of the variance accounted for by the pathway that contains the parts between *M*_*1*_ and *X*_*1*_ and the parts between SWB (*Y*) and *M*_*1*_. The total variance along any particular path equals the product of the variance along different parts of the pathway. In Eq (1), $p_{\left(Y,X_{1}\right)}$ refers to the direct impact while $p_{\left(M_{1},X_{1}\right)}*p_{\left(Y,M_{1}\right)}$ and $p_{\left(M_{2},X_{1}\right)}*p_{\left(Y,M_{2}\right)}$ represent indirect effect. Similarly, the series of this GSEM that depict the contribution of homeownership (*X*_*2*_), self-evaluated household SES (*M*_*1*_) and relative income (*M*_*2*_) to SWB (*Y*) are:

$$r_{\left(Y,X_{2}\right)}=p_{\left(Y,X_{2}\right)}+p_{\left(M_{2},X_{2}\right)}*p_{\left(Y,M_{2}\right)}+p_{\left(M_{1},M_{2}\right)}*p_{\left(Y,M_{2}\right)}$$ (2)

$$r_{\left(Y,M_{1}\right)}=p_{\left(Y,M_{1}\right)}+p_{\left(M_{1},X_{1}\right)}*p_{\left(Y,X_{1}\right)}+p_{\left(Y,X_{2}\right)}*p_{\left(M_{1},X_{2}\right)}$$ (3)

$$r_{\left(Y,M2\right)}=p_{\left(Y,M_{2}\right)}+p_{\left(M_{2},X_{2}\right)}*p_{\left(Y,X_{2}\right)}+p_{\left(M_{2},X_{1}\right)}*p_{\left(Y,X_{1}\right)}$$ (4)

Our analysis strategy is conducted in three stages. First, applying the stepwise analysis with different measures of S-SES as a mediator by using GSEM, we gradually added S-SES variables to explore their different relationship with all respondents’ SWB. All equations have been adjusted using the instrumental variables (IVs) mentioned above, with each IV serving as a regressor for its corresponding endogeneity variable. Second, assuming the underlying determinants on SWB vary from local Hukou individuals (NRs and NLRs) and nonlocal migrants (UUMs and RUMs), we divided the total sample into the four groups mentioned above to estimate the relationship among O-SES, S-SES and SWB. Finally, we explore the mediating effect of S-SES on the relationship between O-SES and SWB among the total sample and each subsample.

## 4. Results

### 4.1 Descriptive statistics

The descriptive statistics of all used variables by total and migration status are demonstrated in Table 1. There were 4,960 valid respondents with 442 migrants and 4,518 local residents. Approximately 58.6% of the total respondents self-reported as happy, and 15% even felt greater. However, there is no significant difference between migrants and local individuals regarding their SWB (*p*-value = 0.466). Table 1 shows that Chinese migrants have significantly higher HIPC than local residents on average, and the maximum value of HIPC of migrants are also much greater than those of locals (2,800,000 vs. 2,000,000). On the contrary, another O-SES indicator homeownership shows a considerable difference between migrants and locals that local workers own their homes much more than migrants (73.9% vs. 39.6%, p<0.01). In terms of S-SES indicators, we do not observe the same significant difference in the self-evaluated household SES between these two groups, yet, those locals have much higher relative income than those migrants on the average and maximum value (p = 0.078).

Except for Hukou status and gender, most of the control variables are statistically “migrant-local” between-group differences. Specifically, local residents are older than migrants and have a higher chance of having more children. Table 1 also shows that the proportion of migrants who are single/separated/divorced is significantly higher than that of local residents. There are also significant differences between local people and migrants in terms of education level, and the proportion of migrants with college/university degrees is higher than that of local individuals (34.8% V.S. 16.9%). We also find the same pattern regarding self-evaluated health status, especially in the “excellent health status” category, verifying the previous arguments of migrants working out are mostly young laborers, so their physical health is generally better [38]. However, in terms of social security coverage and employer ownership, the social security coverage rate of local residents is higher than that of migrants which is consistent with our previous explanation. Additionally, compared with migrants, there are more local residents who work in state-owned enterprises or institutions (9% vs. 13.8%, p = 0.005). This phenomenon may be due to the uneven distribution of resources caused by the system [67, 68]. In examining the macro-level (county or city level) IVs utilized in this study, we find a significant disparity between migrant and local populations concerning average HIPC, homeownership rates, and the percentage of individuals with income at a county or city level. Notably, the average HIPC for migrant households and the percentage of migrants with income substantially exceed those of local households (27031.10 vs. 14289.88 and 91.17% vs. 90.11%). In contrast, homeownership rates are considerably lower among migrant households than local ones. These findings align with the outcomes of our earlier studies on individual-level variables.

### 4.2 Stepwise GSEM

Tables 2 and 3 report the GSEM estimation for the total sample and separate subsample. Table 2 uses a stepwise analysis separately exploring the single effect of two mediators–self-evaluated household SES and the discrepancy between aspiration and absolute income. Thus, Model 1 excludes mediators, while Models 2 through 3 in Table 2 present the corresponding results of different specifications. Finally, the estimations of the full model are shown in Model 4. The model comparison index of -2Log pseudolikelihood, AIC and BIC from Models 1 to 4 show that the model has been improved stepwise.

**Table 2:** Stepwise GSEM estimation.

Dependent variable: SWB	Model 1	Model 2	Model 3	Model 4
***Independent variables***				
*O-SES*				
Household income per capita (HIPC)	0.233***	0.126***	0.231***	0.129***
	(0.035)	(0.030)	(0.032)	(0.028)
Homeownership	0.195***	0.152**	0.194***	0.151**
	(0.072)	(0.072)	(0.072)	(0.072)
*S-SES (Mediators)*				
Household SES		0.619***		0.610***
		(0.051)		(0.051)
Log Relative income			-0.101***	-0.078**
			(0.033)	(0.032)
***Control variables***				
Residential status (refs: migrants)				
New-local residents (NLRs)	0.419*	0.459*	0.400	0.448*
	(0.252)	(0.256)	(0.252)	(0.256)
Native residents (NRs)	0.574**	0.514**	0.565**	0.512**
	(0.254)	(0.258)	(0.254)	(0.257)
Urban areas (refs: rural areas)	0.061	0.142	0.056	0.138
	(0.093)	(0.094)	(0.093)	(0.093)
Hukou (refs: non-agriculture Hukou)	0.***134***	0.090	0.135	0.092
	(0.092)	(0.093)	(0.092)	(0.093)
***Length of residency***	***-0*.*005***	***-0*.*005***	***-0*.*005***	***-0*.*005***
	(0.008)	(0.008)	(0.008)	(0.008)
Length of ***Hukou*** registered	-0.000	0.002	-0.001	0.001
	(0.008)	(0.008)	(0.008)	(0.008)
Employment status (refs: unemployed)				
Farm	-0.082	-0.094	-0.051	-0.071
	(0.190)	(0.189)	(0.192)	(0.190)
Self-employed	0.213	0.110	0.227	0.120
	(0.197)	(0.195)	(0.198)	(0.195)
Employed	0.098	0.090	0.101	0.090
	(0.190)	(0.188)	(0.191)	(0.188)
Works in public sectors (N = 0; Y = 1)	0.037	-0.008	0.035	-0.008
	(0.092)	(0.092)	(0.093)	(0.093)
Log annual working hours	-0.289***	-0.278***	-0.287***	-0.276***
	(0.055)	(0.055)	(0.055)	(0.055)
Age	-0.155***	-0.147***	-0.151***	-0.144***
	(0.025)	(0.025)	(0.025)	(0.025)
Age^2^	0.002***	0.002***	0.002***	0.002***
	(0.000)	(0.000)	(0.000)	(0.000)
Gender	-0.179***	-0.176***	-0.173***	-0.171***
	(0.058)	(0.058)	(0.058)	(0.058)
Married/cohabit	1.048***	0.991***	1.061***	1.003***
	(0.104)	(0.103)	(0.104)	(0.103)
Educational level (refs: illiteracy)				
Compulsory	0.209	0.194	0.208	0.194
	(0.129)	(0.128)	(0.129)	(0.127)
Senior high school	0.197	0.147	0.199	0.149
	(0.147)	(0.146)	(0.147)	(0.146)
College/University	0.340**	0.205	0.347**	0.211
	(0.166)	(0.165)	(0.166)	(0.164)
SRH (refs: poor & worst)				
Neutral	0.293***	0.256**	0.289**	0.254**
	(0.114)	(0.112)	(0.113)	(0.112)
Good	0.761***	0.639***	0.756***	0.636***
	(0.108)	(0.107)	(0.108)	(0.107)
Excellent	1.385***	1.237***	1.378***	1.233***
	(0.116)	(0.116)	(0.116)	(0.116)
Social security	0.226**	0.163	0.222**	0.160
	(0.102)	(0.102)	(0.102)	(0.102)
Number of children alive	0.008	-0.006	0.005	-0.007
	(0.044)	(0.044)	(0.044)	(0.044)
Cut-point 1	-4.627***	-5.289***	-4.619***	-5.244***
	(0.755)	(0.737)	(0.746)	(0.735)
Cut-point 2	-2.936***	-3.583***	-2.925***	-3.535***
	(0.747)	(0.728)	(0.737)	(0.725)
Cut-point 3	-1.478**	-2.096***	-1.465**	-2.046***
	(0.745)	(0.726)	(0.736)	(0.724)
Cut-point 4	1.559**	1.014	1.578**	1.067
	(0.747)	(0.726)	(0.737)	(0.724)
-2Log pseudolikelihood	-14292.44	-18220.09	-21387.86	-25318.00
AIC	28745	36658	42994	50912
BIC	29266	37368	43703	51810
OBS	4960	4960	4960	4960

Notes: 1) SWB: subjective wellbeing, S-SES: subjective socioeconomic status, O-SES: objective socioeconomic status; 2) GSEM analysis with coefficients are reported; 3) household SES as a ranked ordinal variable in GSEM analysis; 4) coefficient results of independent variables and mediators as dependent variables, IVs as explanatory variables are omitted due to limited words but available upon request; 5) AIC = Akaike Information Criterion; BIC = Bayesian Information Criterion; 6) robust standard errors in parentheses, and sig: * p<0.1, ** p<0.05, *** p<0.01.

**Table 3:** GSEM estimation for subsample analysis.

Dependent variable: SWB	Model 4 Full sample	Local subsamples			Migrant subsamples		
		Model 5 All Locals	Model 5a NLRs	Model 5b NRs	Model 6 All Migrant	Model 6a Migrant (U-U)	Model 6b Migrant (R-U)
***O-SES***							
HIPC	0.129***	0.139***	0.072	0.146***	0.073	0.186	0.013
S.D.	(0.028)	(0.031)	(0.071)	(0.035)	(0.078)	(0.171)	(0.130)
Homeowner	0.151**	0.120	-0.030	0.159*	0.450**	0.625**	0.350
S.D.	(0.072)	(0.076)	(0.182)	(0.085)	(0.225)	(0.308)	(0.368)
***S-SES***							
Household SES	0.610***	0.614***	0.583***	0.634***	0.517***	0.311	0.738**
S.D.	(0.051)	(0.054)	(0.134)	(0.059)	(0.187)	(0.234)	(0.313)
Relative income	-0.078**	-0.074**	0.005	-0.089**	-0.131	0.037	-0.329*
S.D.	(0.032)	(0.032)	(0.073)	(0.036)	(0.143)	(0.183)	(0.176)
-2Log pseudolikelihood	-25318.00	-22869.38	-3894.98	-18888.97	-2349.32	-1188.89	-1086.63
AIC	50912	46005	8046	38024	4933	2600	2373
BIC	51810	46858	8643	38790	5411	2983	2707
OBS	4960	4518	783	3735	442	233	209

Data Source: CGSS, 2010.

Notes: 1) HIPC: household income per capita, S-SES: subjective socioeconomic status, O-SES: objective socioeconomic status, NLRs: new-local residents, NRs: native residents, UUMs: urban-to-urban migrants, RUMs: rural-to-urban migrants, HIPC: household income per capita; 2) household SES as a ranked ordinal variable in GSEM analysis; 3) GSEM analysis with coefficients are reported; 4) coefficients of control variables, IVs and mediator variables are omitted due to limited words but available upon requests; 5) AIC = Akaike Information Criterion; BIC = Bayesian Information Criterion; 5) robust standard errors in parentheses, and sig: * p<0.1, ** p<0.05, *** p<0.01.

Model 1 of Table 2 without any mediators explores the pure effect of O-SES on SWB. As CET suggested, the estimations show that HIPC and homeownership could be significantly correlated to respondents’ SWB, supporting Hypothesis 1. In terms of O-SES indicators, for 1% increase in HIPC, the odds of reporting a higher level of SWB increased by 26.23% (exp (0.233) −1 = 0.2623), and respondents own their flat or house were 21.53% more likely to report higher SWB (exp (0.195) −1 = 0.2153), holding all other variables constant. From Model 2 to Model 3, the S-SES variables as mediator is added sequentially. It shows the odds of reporting better SWB decrease when self-evaluated household SES added to Model 2 in terms of those two O-SES indicators. For example, 1% increase in HIPC, the odds of reporting better SWB increased by only 13.43%, and only 16.42% homeowner are more likely to report better SWB, respectively. Based on SCT, self-evaluative household S-SES are significantly and positively associated with SWB among all respondents. Overall, it was found that the higher household S-SES respondents report, the higher odds of reporting better SWB.

Based on ALT, Model 3 presents estimations that take another S-SES into account to explore the association with respondents’ SWB. The results in this model reveal that the effect of the difference between aspiration income and absolute income of themselves negatively contributes to respondents’ SWB, 1% change in relative income, the odds of reporting a better SWB decreased by 9.61%, which is in line with Stutzer’s finding [34].

**Fig 3:**
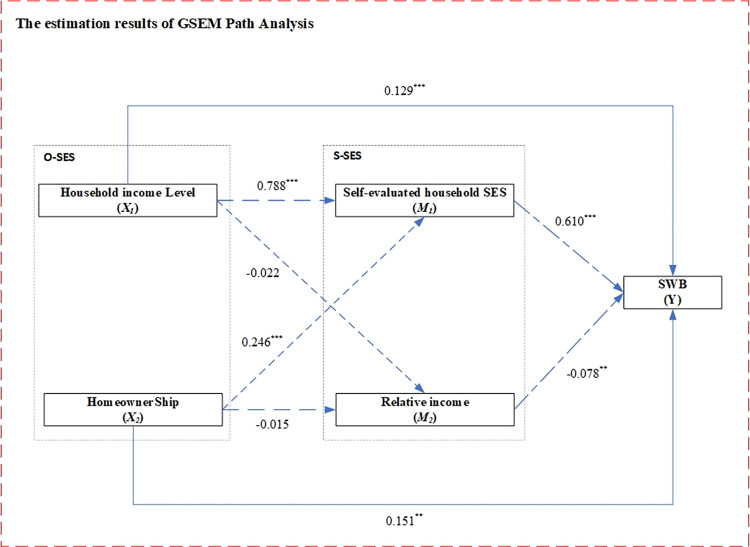
The estimation results of GSEM for full sample. Notes: 1) GSEM: generalized structural equation modeling, O-SES: objective socioeconomic status, S-SES: subjective socioeconomic status, SWB: subjective wellbeing; 2) Solid lines are direct effects and dashed lines are indirect effects; 3) IVs and control variables were included across all analysis, however, their estimated coefficients are omitted as not the focus on this study.

In Model 4 of Table 2, where both O-SES and S-SES are taken into account, most independent variables have slightly changed in the estimate of their correlation with SWB with respect to significance and directions. HIPC and homeownership are positively correlated with SWB, while the discrepancy between aspiration and absolute income of respondents negatively correlated with SWB which verifies Hypothesis 2. However, when considering two S-SES variables, the influence of O-SES, especially homeowner status, its relationship with SWB has changed slightly, which varies from 1% to 5% that verifying Hypothesis 3. These results are also shown in Fig 3.

As the sample is further classified, the subsample analysis is shown in Table 3 and all the models estimate the relationship between O-SES, S-SES and SWB for each subsample. Regarding the impact of O-SES, HIPC is only significantly and positively related to the SWB of local residents (0.139, p<0.01), and homeowner status is also positively related to migrants’ SWB (0.450, p<0.05). Among the local residents, the HIPC and homeownership as O-SES are positively and significantly correlated to NRs’ SWB (0.146, p<0.01; 0.156, p<0.10). For all migrants, only homeownership is positively associated with UUMs’ SWB as we expected (0.618, p<0.05).

In terms of the association between S-SES and SWB for all subsamples, estimations from Model 5a and Model 5b in Table 3 show that household SES as S-SES present a significant and positive correlation with SWB for almost local and migrant subsamples except UUMs. Indicating that most respondents self-feeling the higher the S-SES they evaluated, the higher the level of happiness they reported.

In addition, concerning the other S-SES indicator, i.e., relative income (difference between aspiration income and absolute income) in Table 3 for each subsample, the estimated results from Model 5b and Model 6b present that the relative income is negatively related to NRs’ and RUMs’ SWB at 5% and 10% significance level (-0.089, p<0.05; -0.329, p<0.10), respectively.

### 4.3 GSEM mediation analysis

The GSEM mediation analysis shown in Table 4 indicates the pathway among respondents’ O-SES, S-SES and SWB. Model 4 shows that not only both O-SES indicators (the annual HIPC and homeownership status) on SWB were significantly and directly associated with SWB (0.129, p<0.01 and 0.151, p<0.05), but also other pathways to SWB of these variables have been mediated by self-evaluated household SES (0.481, p<0.01; 0.150, p<0.01). However, we cannot observe any indirect effect through another S-SES indicator of relative income.

**Table 4:** GSEM mediation analysis for S-SES, direct and indirect effects of O-SES on SWB.

Mediators	Direct effect	Indirect effect	
	
		Household SES	Relative income
**Model 4: All sample**			
Household income per capita (HIPC)	0.129***	0.481***	0.002
S.D.	(0.028)	(0.047)	(0.003)
Homeowner	0.151**	0.150***	0.001
S.D.	(0.072)	(0.048)	(0.003)
OBS	4960	4960	4960
**Model 5: Local residents (Full)**			
Household income per capita (HIPC)	0.139***	0.506***	0.002
S.D.	(0.031)	(0.051)	(0.003)
Homeowner	0.120	0.116***	0.001
S.D.	(0.076)	(0.051)	(0.003)
OBS	4518	4518	4518
**Model 5a: New-local residents (NLRs)**			
Household income per capita (HIPC)	0.072	0.415***	0.000
S.D.	(0.071)	(0.111)	(0.001)
Homeowner	-0.030	0.164	-0.001
S.D.	(0.182)	(0.114)	(0.007)
OBS	783	783	783
**Model 5b: Native residents (NRs)**			
Household income per capita (HIPC)	0.146***	0.542***	0.004
S.D.	(0.035)	(0.058)	(0.004)
Homeowner	0.159*	0.116***	-0.001
S.D.	(0.085)	(0.058)	(0.004)
OBS	3735	3735	3735
**Model 6: Migrants (Full)**			
Household income per capita (HIPC)	0.073	0.234***	-0.006
S.D.	(0.078)	(0.108)	(0.017)
Homeowner	0.450**	0.425**	0.015
S.D.	(0.225)	(0.194)	(0.022)
OBS	442	442	442
**Model 6a: Urban-to-urban migrants (UUMs)**			
Household income per capita (HIPC)	0.186	0.104	-0.003
S.D.	(0.171)	(0.097)	(0.015)
Homeowner	0.625**	0.222	-0.011
S.D.	(0.308)	(0.198)	(0.053)
OBS	233	233	233
**Model 6b: Rural-to-urban migrants (RUMs)**			
Household income per capita (HIPC)	0.013	0.485**	-0.011
S.D.	(0.130)	(0.243)	(0.053)
Homeowner	0.350	0.687*	-0.011
S.D.	(0.368)	(0.379)	(0.053)
OBS	209	209	209

Notes: 1) GSEM mediation analysis for S-SES; 2) control variables and IVs are included in each model, yet the results are omitted due to word limitation; 3) household SES as a ranked ordinal variable in GSEM analysis; 4) standard errors in parentheses, sig: * p<0.1, ** p<0.05, *** p<0.01.

The subsample estimation tells a different story. Model 5, Models 5a and Model 5b are local subsamples, and the latter two have different residency lengths. The estimated results of Model 5b present that HIPC and homeowner status in urban city matter directly and indirectly on NRs’ SWB, particularly through those with higher self-evaluated household S-SES. Moreover, the relationship between HIPC and SWB for NLRs (Model 5a) was mediated by household S-SES measures, yet it had no direct relationship with their SWB.

In terms of migrant subsamples, Model 6 shows that whether one owns a house or apartment in the host location (i.e., homeowner status) expresses a significant direct relation with all migrants’ SWB (0.45, *p* < 0.05) and through household S-SES with a significant indirect effect (0.234, *p* < 0.05; 0.425, *p* < 0.05). Moreover, the household S-SES at a high-level positively mediated the relationship between HIPC and SWB as well as homeownership and SWB among RUMs’ subsample (Model 6b), which is consistent with our argument above. Overall, such findings verify Hypothesis 4 that the mediating effect of S-SES on the relationship between O-SES and SWB varies between locals and migrants.

## 5. Discussion

### 5.1 Theoretical implications

This study contributes to previous research by taking multiple measurements of S-SES into account simultaneously and considering the mediating effect of S-SES on the relationship between O-SES and SWB. In addition, the NLRs in local Hukou respondents and UUMs in migrants who are usually overlooked by other researchers before are also considered in our study. Therefore, the total sample is divided into NLRs, NRs, UUMs and RUMs.

Regarding S-SES, the results from GSEM estimation demonstrate that household SES is positively correlated to NRs and NLRs’ SWB. These findings may be because RUMs’ motivation for migration is to earn more money to improve the living standard of the family members left-behind [37]. The prevalence of empty-nest elders and left-behind children in rural China proves that many RUMs couples go to another city to work together to earn money and improve their living conditions [69]. The most crucial issue that RUMs are concerned about is whether the income can build a house in their hometown or the living conditions of family members in their hometown can be significantly improved [69]. Therefore, RUMs are concerned about the status of the whole family even more. In addition, in terms of the GSEM mediation analysis, the estimations for local residents have indicated that household income and homeownership as O-SES is positively related to the SWB in direct and indirect ways on NRs through the mediating effect of household SES. However, HIPC as an indicator of O-SES plays no direct relationship with NLRs’ SWB. The relationship between HIPC and SWB for NLRs is significantly mediated by the self-evaluated household SES. Moreover, regarding the estimations for the migrant sample, HIPC has neither a direct nor indirect association with SWB for UUMs, while homeownership only has indirect relation with UUMs’ SWB. The relation between SWB, HIPC and homeownership is significantly and positively mediated by the high-level of self-evaluated household SES (S-SES). Overall, in light of the pathway analysis for each group, it is concluded that HIPC as a typical index of O-SES has no direct correlation with the SWB of all migrants and NLRs, which is supported by the theories of SCT and ALT. In addition, the self-evaluated household SES as S-SES plays a major mediating role in the relation between SWB and O-SES. This may be attributed to all migrants and NLRs who have left their birthplaces being more concerned about whether the host city has accepted them. No matter what ambitions or aspirations, the SWB would mainly rely on the comparison psychology in China. Consequently, their psychological emotions, acceptance and sense of belonging contribute more to SWB instead of the actual earnings.

With respect of the other important indicator of S-SES in our mediating effect model, i.e., the difference between aspiration income and absolute income, the GSEM estimation results show that even if UUMs are driven by a great ambition to migrate to metropolitan cities, their ambitions and aspirations is not significantly related to SWB. Realizing the discrepancy between aspired status and current status does not significantly determine all migrants’ and NLRs’ SWB. In addition, results from GSEM mediation analysis have shown that the discrepancy between one’s aspiration and absolute income as S-SES does matter for the relationship between all respondents’ SWB and O-SES (HIPC and homeownership).

Such findings indicate that respondents, especially migrants and NLRs, are not more concerned about their current status (O-SES) or whether their aspirations and ideals could be realized which contributes to previous literature that an individual’s ideals and aspirations do not necessarily determine their SWB. This also reflects the different cultural environments between China and other countries. Chinese people pay more attention to the results after comparison with others. Specifically, they are more concerned about whether their current status is much better than others (social comparison), and whether they are wealthy or have a higher socioeconomic status than other people [5, 60, 70]. The original aspirations (original ideals) that they initially hold are often left-behind. Additionally, the insignificant mediation effect of the difference between aspiration income and absolute income on the relationship between SWB and O-SES may be attributed to their aspirations that have been climbing as the environment changes. According to the “hedonic treadmill hypothesis”, aspirations will adapt to the new circumstance [70]. Therefore, most people may be aware of human nature’s character that realizing the current target or aspirations cannot generate the sense of happiness for a long time as they will have new pursuits further [71]. On the other hand, as ALT suggested, a sudden increase in income may have arrived at the prior aspiration level, which brings favorable impacts for a short period [71]. For example, winning a lottery can realize short-term economic requirements and improve the quality of living status with enormous pleasure, but people will adapt to the comfort of new wealth soon and desire to have other things (e.g., social position or healthy).

### 5.2 Limitations and directions for future research

This study, while endeavoring to deliver meaningful insights, is not without its limitations. First, we were only able to garner information on the crucial independent variable, income aspiration, from the CGSS 2010 dataset. Regrettably, we were unable to locate this specific form of income aspiration in other microlevel large secondary datasets. Additionally, in consideration of the rapid urbanization prior to 2012, as well as the need to circumvent the impact of the housing restriction policy enacted post-2012 in China, we found it necessary to utilize data from the 2010 survey wave even we could face with the insufficient data timeliness. Second, our focus on working individuals aged between 16 and 60 resulted in a migrant sample size of 442 observations, which can be considered relatively small. Despite the nationally representative nature of our data and the post-hoc power analysis results, which suggest an 80% probability of detecting a true effect in our analysis, thereby reinforcing the adequacy of our sample size, we acknowledge that a larger sample size could potentially yield more compelling evidence. Finally, it should be noted that this paper predominantly tackles a correlation issue as opposed to a causal one, even though we implemented the IV-GSEM approach. Specifically, our objective was to explore the relationship framework or pathway between disparate representative factors under the umbrella of CET, SCT, and ALT, i.e., O-SES, S-SES, and SWB, and to verify the stability of these relationships. However, owing to the constraints imposed by the dataset, addressing these limitations within this study proved challenging, which underscores the need for further comprehensive research.

## 6. Conclusion

Overall, this study has analyzed the pathway on the mediating effect of S-SES on the relationship between SWB and O-SES using 2010 CGSS. The empirical analysis concluded that household income and homeownership as O-SES show a positive relationship with SWB and the self-evaluated household SES as S-SES plays a decisive role in mediating the relationships between SWB and O-SES, which provides useful evidence to policymakers to consider how to mitigate the negative impacts. Regarding the over-comparison and ideals oblivion in China, it is necessary to establish correct values for children and young people by influencing ideological education and spiritual civilization. It is also possible to stimulate several public media platforms to propagandize correct values to reduce people’s mentality of overemphasizing material comparisons and vanity. Additionally, regarding RUMs who prefer to send earnings back to their hometowns, the government could try to provide some low-rent houses with acceptable standards in large cities, living communities, community care activities and other welfare policies to improve RUMs’ life quality and help them feel warmth and respect in metropolitan cities. Above findings not only exposed the status of SWB in China, but also revealed the cultural limitations and applicability of the point that aspiration had an important impact on SWB which was put forward by early Western researchers. Our findings confirm that the notion that aspirations have great contribution to SWB is not globally applicable, at least not for a country like China. Therefore, when we explore the determinant factors of SWB, it is necessary to carry out the targeted research on different groups and cultural backgrounds.

## Supporting information

S1 File (ZIP)

## List of abbreviations

AIC: Akaike Information Criterion
ALT: Aspiration Level Theory
ANOVA: Analysis of variance
BIC: Bayesian Information Criterion
CET: Classical Economic Theory
CGSS: China General Social Survey
GSEM: Generalized structural equation modeling
HIPC: Household income per capita
NLRs: New-local residents
NRs: Native residents
O-SES: Objective Socioeconomic Status
RUMs: Rural-to-urban migrants
SCT: Social Comparison Theory
SES: Socioeconomic Status
SES: Self-evaluated socioeconomic status
SRH: Self-rated health
S-SES: Subjective Socioeconomic Status
SWB: Subjective Wellbeing
UUMs: Urban-to-urban migrants
WTO: World Trade Organization
